# Amino Acid Substitutions in Genotype 3a Hepatitis C Virus Polymerase Protein Affect Responses to Sofosbuvir

**DOI:** 10.1053/j.gastro.2019.05.007

**Published:** 2019-09

**Authors:** Peter A.C. Wing, Meleri Jones, Michelle Cheung, Sampath DaSilva, Connor Bamford, Wing-Yiu Jason Lee, Elihu Aranday-Cortes, Ana Da Silva Filipe, John McLauchlan, David Smith, William Irving, Morven Cunningham, Azim Ansari, Eleanor Barnes, Graham R. Foster

**Affiliations:** 1 Barts Liver Centre, Blizard Institute, Queen Mary University of London, London, UK; 2 Medical Research Council, University of Glasgow Centre for Virus Research, Glasgow, UK; 3 Nuffield Department of Medicine, University of Oxford, Oxford, UK; 4 National Institute for Health Research, Oxford Biomedical Research Centre, Oxford University National Health Service Trust, Oxford, UK; 5 National Institute for Health Research, Nottingham Biomedical Research Centre, Nottingham University Hospitals National Health Service Trust, Nottingham, UK; 6 University of Nottingham, Nottingham, UK

**Keywords:** mutation, amino acid change, genetics, response to therapy, CI, confidence interval, DAC, daclatasvir, HCV, hepatitis C virus, IC_50_, 50% inhibitory concentration, IFN, interferon, RBV, ribavirin, SOF, sofosbuvir, SVR, sustained virologic respone, Wt, wild-type

## Abstract

**Background & Aims:**

Sofosbuvir is a frequently used pan-genotype inhibitor of hepatitis C virus (HCV) polymerase. This drug eliminates most chronic HCV infections, and resistance-associated substitutions in the polymerase are rare. However, HCV genotype 3 responds slightly less well to sofosbuvir-based therapies than other genotypes. We collected data from England’s National Health Service Early Access Program to search for virus factors associated with sofosbuvir treatment failure.

**Methods:**

We collected patient serum samples and used the capture-fusion assay to assess viral sensitivity to sofosbuvir in 14 HCV genotype 3 samples. We identified polymorphisms associated with reduced response and created modified forms of HCV and replicons containing the substitutions of interest and tested their sensitivity to sofosbuvir and ribavirin. We examined the effects of these polymorphisms by performing logistic regression multivariate analysis on their association with sustained virologic response in a separate cohort of 411 patients with chronic HCV genotype 3 infection who had been treated with sofosbuvir and ribavirin, with or without pegylated interferon.

**Results:**

We identified a substitution in the HCV genotype 3a NS5b polymerase at amino acid 150 (alanine [A] to valine [V]), V at position 150 was observed in 42% of patients) with a reduced response to sofosbuvir in virus replication assays. In patients treated with sofosbuvir-containing regimens, the A150V variant was associated with a reduced response to treatment with sofosbuvir and ribavirin, with or without pegylated interferon. In 326 patients with V at position 150, 71% achieved an sustained virologic response compared to 88% with A at position 150. In cells, V at position 150 reduced the response to sofosbuvir 7-fold. We found that another rare substitution, glutamic acid (E) at position 206, significantly reduced the response to sofosbuvir (8.34-fold reduction); the combinations of V at position 150 and E at position 206 reduced the virus response to sofosbuvir 35.77-fold. Additionally, in a single patient, we identified 5 rare polymorphisms that reduced sensitivity to sofosbuvir our cell system.

**Conclusions:**

A common polymorphism, V at position 150 in the HCV genotype 3a NS5b polymerase, combined with other variants, reduces the virus response to sofosbuvir. Clinically, infection with HCV genotype 3 containing this variant reduces odds of sustained virologic response. In addition, we identified rare combinations of variants in HCV genotype 3 that reduce response to sofosbuvir.

**What You Need to Know**

**Background and Context**

Patients with genotype 3 HCV respond less well to therapy with direct-acting antiviral agents and the response is greatly reduced in patients with cirrhosis, who often have fewer retreatment options. The mechanisms for this reduced response are unclear.

**New Findings**

The authors identified polymorphisms in genotype 3 HCV (A150V and K206E) that reduce the response to sofosbuvir. The A150V variant is common and K206E variant is rare. Additional very rare polymorphisms reduce the response in combination.

**Limitations**

Additional study is needed on the role of this polymorphism in disease progression and sensitivity to triple therapy

**Impact**

The authors identified a polymorphism in HCV genotype 3 that affects virus response to treatment. This discovery could increase our understanding of mechanisms of treatment failure and might change treatment regimens for patients with HCV genotype 3 infection.

Sofosbuvir (SOF) is a highly effective antiviral drug that has replaced interferon (IFN) as the backbone of many therapies for patients with chronic hepatitis C virus (HCV) infection.^1^ In combination with drugs targeting other viral enzymes (including the poorly potent guanosine analogue, ribavirin [RBV],^2^ or highly potent inhibitors of viral NS5a^3^ or protease proteins^4^) most patients clear virus, and resistance polymorphisms known to reduce SOF’s antiviral effects are almost never observed,^5^ allowing effective retreatment.^6^ The mechanism of treatment failure in the small proportion of individuals who do not respond remains unknown. The recent introduction of triple-therapy regimens (SOF with a pan-genotypic protease and NS5a inhibitors^7^, ^8^) provides an alternative to the current approach to therapy, which combines SOF with an NS5a inhibitor alone.^3^, ^9^ However, protease inhibitors are contraindicated in patients with decompensated cirrhosis, and in such patients, the only available therapy is SOF plus an NS5a inhibitor. It is not known which patients require triple therapy and it is not clear whether this regimen should be reserved as a retreatment option for the few patients who do not respond to dual therapy.

HCV genotype 3a is common in the Indian sub-continent and comprises 30% of all HCV infections in Eastern and Western Europe.^10^ Patients infected with this genotype respond well to IFN-based regimens, with response rates approaching 80% in patients without cirrhosis, although the response is greatly reduced in those with advanced fibrosis.^11^, ^12^ SOF is active against HCV genotype 3 and in combination with the pan-genotypic NS5a inhibitor velpatasvir,^9^ >90% of patients respond. However, response rates with SOF/velpatasvir are lower in patients with cirrhosis than in those with lesser degrees of fibrosis and the response to therapy is further reduced in patients previously exposed to IFN-based therapies. For patients with decompensated cirrhosis, for which protease inhibitors are contraindicated, response rates for patients with genotype 3 may be as low as 50%.^8^ It is unclear why HCV genotype 3 is less sensitive to SOF than other HCV genotypes, and it is not known why prior therapy with IFN impacts response.

The extraordinary success of SOF-based treatments in both clinical trials and real-world studies, with sustained virologic response (SVR) rates of >95%, has reduced the opportunity to study patients who do not respond—there are very few patients to examine. Viral sequencing of this highly polymorphic, rapidly evolving virus has, to date, not identified motifs clearly associated with a reduced response to SOF. In vitro, a variant at position 282 (S282T polymorphism) is commonly selected, although it does have a reduced replication capacity.^13^ In clinical studies, the S282T variant is rarely seen^14^ and the “resistance polymorphisms” that have been reported after antiviral therapy (L159F and V321A) do not appear to reduce the effectiveness of SOF in viral replication models.^15^ Studies identifying treatment outcome of pre-existing resistance variants in baseline clinical samples show that presence of variants susceptible to treatment can dilute the effect of resistance variants to therapy.^16^ This suggests that polymorphisms that reduce response to SOF will be difficult to detect by sequence analysis alone. A more informative approach would be to combine high-throughput sequencing data with in vitro antiviral phenotyping of clinical isolates to identify rare motifs that impact viral response to SOF and other potent antiviral agents.

To address the issue of “SOF resistance,” we studied patients from the National Health Service England Early Access Programme,^17^, ^18^ which offered antiviral therapy with SOF, RBV, and NS5a inhibitors (either daclatasvir [DAC] or ledipasvir) to patients with advanced liver disease. The program was initiated before drug licensing and used treatment durations for HCV genotype 3 infection that, in retrospect, may have been suboptimal, that is, 12 weeks, rather than the now recommended 24 weeks. The high relapse rate (approximately 30%) in patients with HCV genotype 3 prompted a search for viral factors associated with SOF treatment failure.

## Methods

### Study Samples

Serum and plasma samples from patients who donated samples to the HCV Research UK Biobank were used after informed consent and approval by the Biobank Tissue Access Committee, as approved by a UK Ethical Review Committee. Additional samples were obtained after informed consent and Ethics Committee approval from patients attending the Royal London Hospital.

### In Vitro Analysis

Viral phenotyping was performed using the “capture-fusion” viral replication assay in which infected serum is incubated with monocytes before fusion of HCV containing monocytes with hepatocytes.^19^ Library preparation, sequencing, assembly, and variant detection are described in the Supplementary Methods.

### Site-Directed Mutagenesis and Transfection Hepatitis C Virus Replicon Constructs

Nucleotide changes were introduced into the genotype 3 S52-(SHI) sub-genomic replicon^1^ (a kind gift from Charlie Rice), and the full-length genotype 3 DBN (a kind gift from Jens Bukh)^19^ clone using the Quick-Change II XL site-directed mutagenesis kit (Agilent, Santa Clara, CA). Nucleotide changes were confirmed by Sanger sequencing (Cambridge Bioscience, Cambridge, UK). Replicon plasmids were linearized with *XbaI*, treated with mung bean nuclease, purified, and in vitro–transcribed using the MEGAscript T7 Transcription kit (Ambion, Austin, TX). For electroporation, cells were washed twice with ice-cold phosphate buffered saline and diluted to a concentration of 5 × 10^6^ cells/mL. Five micrograms of purified RNA was mixed with 400 μL cell suspension and electroporated using a BioRad Genepulser II on the exponential setting (pulse settings: 250V and 950 μF). To generate the stable replicon cells, cells were grown with G418 (750 μg/mL). For antiviral drug assays, cells were transferred to complete medium and seeded into 96-well plates and left to recover for 24 hours. Antiviral drugs were added for 72 hours, after which cells were lysed in 1X passive lysis buffer and firefly luciferase activity was measured as per manufacturer’s instructions (Promega, Madison, WI).

### Generation of Infectious Genotype 3 Derived Cell Culture Hepatitis C Virus

Huh7.5-SEC14L2 cells were seeded in a 6-well plate at a density of 4.2 × 10^5^ cells/well 24 hours before transfection. Lipofectamine 3000 (3.75 μL) was diluted in 125 μL of OptiMEM and 5 μg of DBN3acc RNA was diluted in 250 μL of OptiMEM. Both mixes were incubated at room temperature for 5 minutes before combining, after which the reaction mixture was incubated for an additional 20 minutes at room temperature and then added drop wise to the cells. After 24 hours, cells were placed in Dulbecco’s modified Eagle medium 10% fetal calf serum and expanded into T25-cm^2^ then T75-cm^2^ flasks. At 7 days post transfection, cells were seeded onto coverslips to assess presence of HCV replication complexes by NS5a immunofluorescence. Once >50% of cells were deemed to be positive for HCV, supernatants from the transfected cells were filter-sterilized using a 0.45-μm syringe filter, aliquoted, and stored at –80°C.

### Statistical Analysis

Multivariate logistic regression was used for the analysis of association between SVR and the viral polymorphisms A150V and K206E and to test for interaction between the 2 sites and its impact on the SVR. To account for population structure of the host and the virus, we performed principal component analysis on host genome-wide genotype data and on virus whole genome nucleotide data. Two virus principal components and 3 host principal components were used as covariates in the model to account for the host and virus population structures. We also added patients’ *IFNL4* genotypes (CC vs non-CC), cirrhosis status, and previous IFN-based treatment status in our model as covariates to account for possible confounding. The multiple comparisons in the capture fusion assay experiments were analyzed using Kruskal-Wallis analysis and changes in drug sensitivity in replication assays were determined to be statistically significant if the 95% confidence intervals (CIs) did not overlap.

## Results

### Viral Phenotyping Reveals Distinct Patterns of Polymorphisms in Patients With a Reduced Response to Sofosbuvir

**Figure 1:**
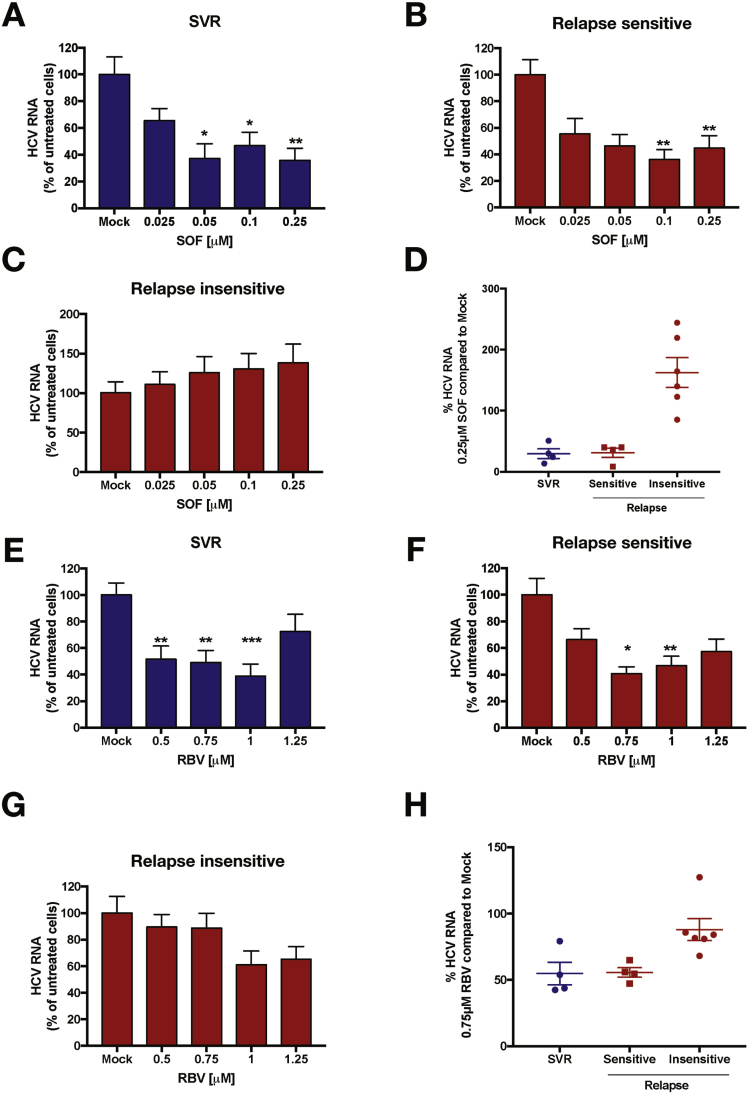
SOF sensitivity in HCV genotype 3 treatment non-responders to direct-acting antiviral (DAA) therapy was assessed using the capture-fusion assay. Sera from patients with HCV genotype 3 (n = 14) who achieved SVR (*blue*) or relapsed (*red*) were used to assess sensitivity to SOF and RBV. Changes in HCV RNA for SOF and RBV in patients who achieved SVR (n = 4) (*A, E*) or relapsed are shown. Samples from patients who relapsed were further divided into 2 groups, depending on the assay outcome, those who were SOF- and RBV-sensitive (n = 4) (*B, F*) and insensitive (n = 6) (*C, G*). Data were summarized in (*D*; SOF) and (*G*; RBV) to show HCV RNA as a percentage of no drug treatment for a single dose of drug (0.25 μM of SOF and 0.75 μM of RBV). Graphs show mean ± SEM. *P* values were calculated using Kruskal-Wallis test. Drug sensitivity of each sample was assessed in quadruplicate for each concentration.

**Supplementary Figure 1:**
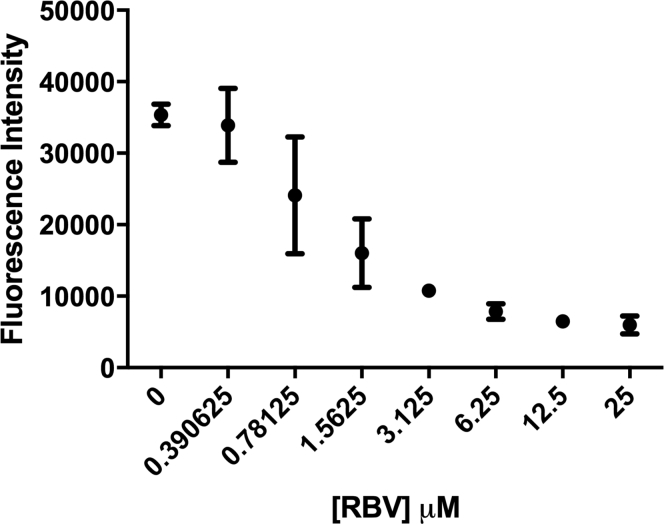
Huh7.5 cells were incubated with a serial dilution of RBV for 72 hours in triplicate. AlamarBlue reagent was added for 3 hours and fluorescence intensity was assessed as a measure of cytotoxicity.

We used the capture-fusion assay^19^ to assess SOF and RBV sensitivity of 14 HCV genotype 3 samples. These included pretreatment viral samples sourced from patients with advanced liver disease from the National Health Service England Early Access Programme (n = 10) and 4 additional HCV genotype 3 samples from patients who had either responded to therapy in the early access program (n = 2) or had previously shown a response to SOF in vitro (n = 2) and were later treated successfully. Samples from SOF-treated patients were selected on the basis of viral load (>1 × 10^5^ IU/mL) and availability (Supplementary Table 1). Sensitivity of samples was assessed by treatment of infected cells with a range of SOF and RBV doses (0–0.25 μM and 0–1.25 μM, respectively) and measurement of HCV RNA in response to drug treatment (SOF, Figure 1*A–D* and RBV, Figure 1*E–G*). These analyses were performed without prior knowledge of treatment outcome. After unblinding, drug sensitivity data for all samples tested were grouped into treatment outcome. Samples from patients who achieved SVR demonstrated a significant reduction in HCV RNA in response to treatment with SOF (Figure 1*A*) and RBV (Figure 1*E*). Note that in the capture-fusion assay, residual signal leads to apparent detection of virus even at high doses of inhibitor and hence viral replication does not fall to zero. Data obtained from samples from patients who relapsed showed 3 distinct patterns of response to SOF. We defined these as “relapse sensitive” or “relapse insensitive,” based on an examination of the dose–response curves. Significant reductions in HCV RNA were only seen in 4 samples in response to SOF and RBV (Figure 1*B* and *F*, “sensitive” sample), while the majority of samples from patients who relapsed (n = 6) exhibited no substantial change in HCV RNA when treated with a range of SOF and RBV doses (“insensitive” samples). To highlight the different drug sensitivities between the sample groups, we analyzed the percentage change in HCV RNA compared to the no-drug control at individual doses of 0.25 μM SOF (Figure 1*D*) and 0.75 μM RBV (Figure 1*H*). A mid-range dose of RBV was selected for the comparison due to some cytotoxicicty at high RBV concentrations (see Supplementary Figure 1). Four viral samples from patients who relapsed showed a comparable reduction in HCV RNA to samples from patients who achieved SVR, while 6 of the 10 samples from patients who relapsed showed no change in HCV RNA with SOF or RBV treatment at this dose.

Samples were subjected to high-throughout, next-generation sequencing with substitutions present in <15% of the sequencing reads discarded. Viral sequences were then compared to the reference genotype 3a sequence (NC_004102) (Table 1). No reported SOF resistance associated substitutions (L159F, S282T, or V321A^20^) were found, but 5 of 6 SOF “insensitive” samples had polymorphisms in domains 1 and 2 of the polymerase protein—A150V and K206E—that were not seen in combination in the sensitive samples, although the individual substitutions were observed. The other SOF-insensitive patient had 5 changes, 4 of which were unique (A150S, G188D, T213N, and N244I).

**Table 1:** Identified Polymorphisms Within the NS5B of the Phenotypes Samples

Patient no.	Therapy	Therapy outcome	Assay outcome (SOF)	Drug sensitivity		Relevant polymorphisms, *%* (pre→post therapy)						
				SOF IC_50_, *μM*	RBV IC_50_, *μM*	K100R	A150V	A150S	G188D	K206E	T213N	N244I
1	SOF/DAC/RBV	SVR	Sensitive	0.039	0.786	—	V (98)	—	—	—	—	—
2	SOF/VEL	SVR	Sensitive	0.034	0.545	—	—	—	—	—	—	—
3	SOF+OBV /PTV/RBV	SVR	Sensitive	0.011	0.419	—	—	—	—	—	—	—
4	SOF/DAC	SVR	Sensitive	0.054	0.19	—	—	—	—	E (98)	—	—
5	SOF/LDV/RBV	Relapse	Sensitive	0.06	0.43	—	—	—	—	E (99)	—	—
6	SOF/DAC/RBV	Relapse	Sensitive	0.033	0.886	—	—	—	—	E (99)	—	—
7	SOF/LDV/RBV	Relapse	Sensitive	0.04	0.72	—	V (98)	—	—	—	—	—
8	SOF/LDV	Relapse	Sensitive	0.02	0.701	—	—	—	—	—	—	—
9	SOF/DAC/RBV	Relapse	Insensitive	>0.25	>1.25	R (65→98)	—	S (98–99)	D (98→97)	—	N (98→99)	I (94–99)
10	SOF/LDV/RBV	Relapse	Insensitive	>0.25	>1.25	R (97→98)	V (54–98)	—	—	E (89→99)	—	—
11	SOF/LDV/RBV	Relapse	Insensitive	>0.25	>1.25	—	V (98–95)	—	—	E (98→99)	—	—
12	SOF/DAC/RBV	Relapse	Insensitive	>0.25	>1.25	—	V (98–97)	—	—	E (100→99)	—	—
13	SOF/LDV/RBV	Relapse	Insensitive	>0.25	>1.25	—	V (98–99)	—	—	E (98→99)	—	—
14	SOF/LDV/RBV	Relapse	Insensitive	>0.25	>1.25	—	V (99)^a^	—	—	E (100)^a^	—	—

NOTE. Patients were divided into 3 groups (SVR-sensitive, relapse-sensitive, and relapse-insensitive) based on therapy and assay outcomes. Samples were deemed to be SOF-insensitive if a single dose of the antiviral drug (0.25 μM) did not reduce the HCV RNA levels to <80% of the no-drug control in the capture fusion assay. Polymorphisms were identified by comparison of NS5b next-generation sequencing data from drug-sensitive vs drug-insensitive samples. The majority of the polymorphisms were identified in the assay-insensitive group, with a specific pattern (A150V and K206E) seen in patients 10–14. Percentage values indicate the frequency of each polymorphism present in the viral populations.

LDV, ledipasvir.

a No post-treatment sequencing data were obtained for sample 14 due to low viral load post relapse.

### Prevalence and Impact of NS5b Mutations in a Second Patient Cohort

**Supplementary Figure 2:**
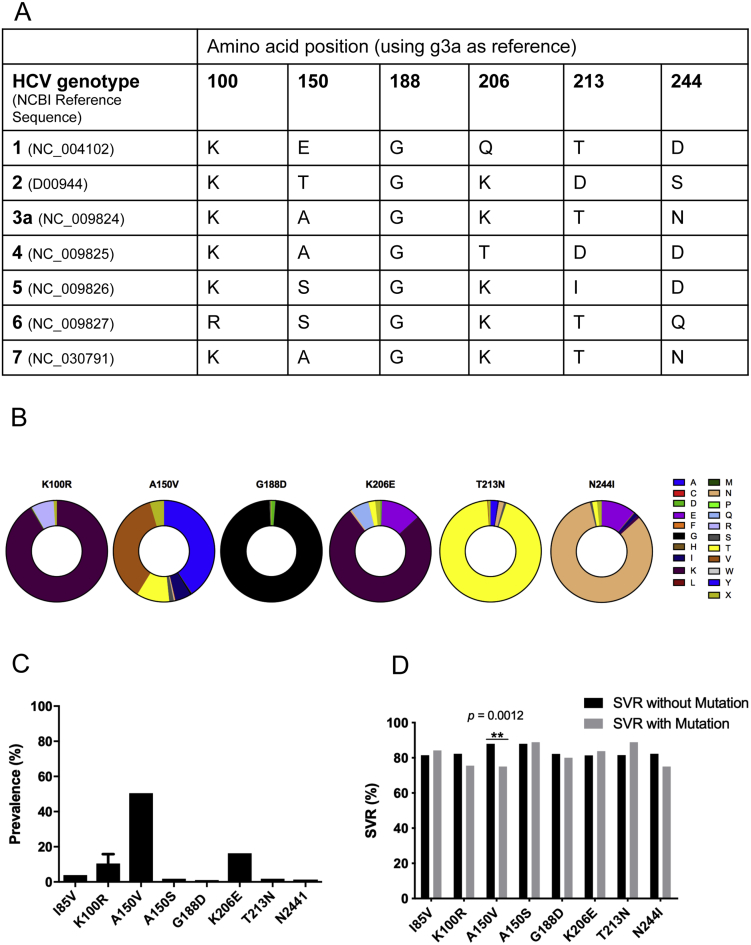
(*A*) HCV genotype 1–7 amino acid sequences were aligned and the table shows the amino acid at the positions of interest and compared to the gt3a reference sequence. (*B*) Alignment of 1200 HCV genotype 3a sequences from the HCV-GLUE database identifying the minor variants at each position of interest. *Legend* designates the color code for each amino acid. (*C*) Analysis of the NS5b polymorphisms in the BOSON cohort, the prevalence of the NS5b polymorphisms was assessed from >500 viral sequencing samples obtained from the BOSON cohort. (*D*) Each mutation was tested to assess whether the frequency was significantly different between SVR and non-SVR samples. Frequencies were analyzed using Fisher’s exact test.

The reference sequences of the 7 HCV genotypes were aligned and the amino acids at the polymorphisms of interest were compared (Supplementary Figure 2*A*). Positions 150, 213, and 244 appeared to be the least conserved across the genotypes. Alignment of 1200 HCV genotype 3a sequences from the HCV-GLUE database (a UK-based database of published HCV sequences)^21^ (amino acids 91–250) (Supplementary Figure 2*B*) showed that amino acids 150 and 206 were the least conserved of the positions of interest and that amino acid 150, in particular, is highly polymorphic. In comparison, available sequences for HCV genotype 3b in HCV-GLUE shows that alanine at position 150 is dominant (n = 39/42). In HCV genotype 3a sequences, alanine at position 150 is present at a frequency of 40.65%, with the valine variant present at a frequency of 36.66%. The variation at position 206 is much less pronounced, with lysine (K) dominating at a frequency of 76.31% and glutamic acid (E) seen at a frequency of only 12.74%. An analysis of the combination of both polymorphisms, alanine at position 150 and glutamic acid at position 206 (A150V_K206E), was present in <4% of the population, making this a rare combination.

We examined the impact of the polymorphisms of interest in pretreatment samples from a cohort of HCV genotype 3 patients who were treated using SOF and RBV, with or without pegylated IFN (BOSON trial).^22^ In this cohort, the frequency of the polymorphisms seen in patient 9 (above) were too low to provide meaningful data (individually all were present in <10% of the population and combinations were present in fewer than 10 patients), but A150V and K206E polymorphisms were common (Supplementary Figure 2*C*). We investigated the association between A150V and K206E amino acid substitutions and outcome (Supplementary Figure 2*D*). To limit the impact of population structure and host genetics, we investigated patients with self-reported white ancestry and genotype 3a virus (n = 411 patients). At site 150 in NS5b protein, the most commonly observed amino acids were valine (42% = 170/407) and alanine (38% = 156/407). We also observed threonine (n = 13%), isoleucine (4%), serine (1.5%), glycine (1%), and asparagine (0.5%). As valine was the most common variant identified in our phenotypic studies, we analyzed isolates carrying valine or alanine at this site (n = 326). In logistic regression multivariate analysis accounting for population structure of virus and host (using principal component analysis), host *IFNL4* genotype (CC vs non-CC), cirrhosis status, and previous treatment status, the polymorphism A150V was associated with an increase in relapse (*P* = .0027; SVR in patients whose virus carries alanine at position 150 = 88% [n = 138/156] and valine at position 150 = 71% [n = 121/170]) (Supplementary Figure 1*C*).

At site 206 in NS5b, the most commonly observed amino acids were lysine at 79% (n = 323/410) and glutamic acid at 13% (n = 54/410). We also observed glutamine (5%), threonine (2.4%), and, rarely, asparagine and aspartic acid. We used the same logistic regression model and included isolates carrying lysine or glutamic acid for analysis (n = 377). We did not observe an association between the polymorphism K206E and treatment outcome (*P* = .26; SVR in patients whose virus carries K206 = 79% [n = 255/323] and E206 = 83% [n = 45/54]).

To look at the interactions among the 3 sites, we included isolates carrying A or V at site 150 and K or E at site 206 in NS5b protein (AE = 25, AK = 120, VE = 16, and VK = 139). Using the same multivariate logistic regression, including an interaction term between the 2 polymorphisms A150V and K206E, we observed no significant interaction between the 2 substitutions (*P* = .13). Analyzing the haplotypes, we observed the lowest rate of SVR in patients whose virus carries the VK haplotype at 68% (n = 95/139), while the other haplotype carriers all had higher rates of SVR (AK = 89% [n = 107/120], AE = 88% [n = 22/25], and VE = 81% [n = 13/16]).

### Structural Relevance of the Identified Polymorphisms in Hepatitis C Virus NS5b

**Figure 2:**
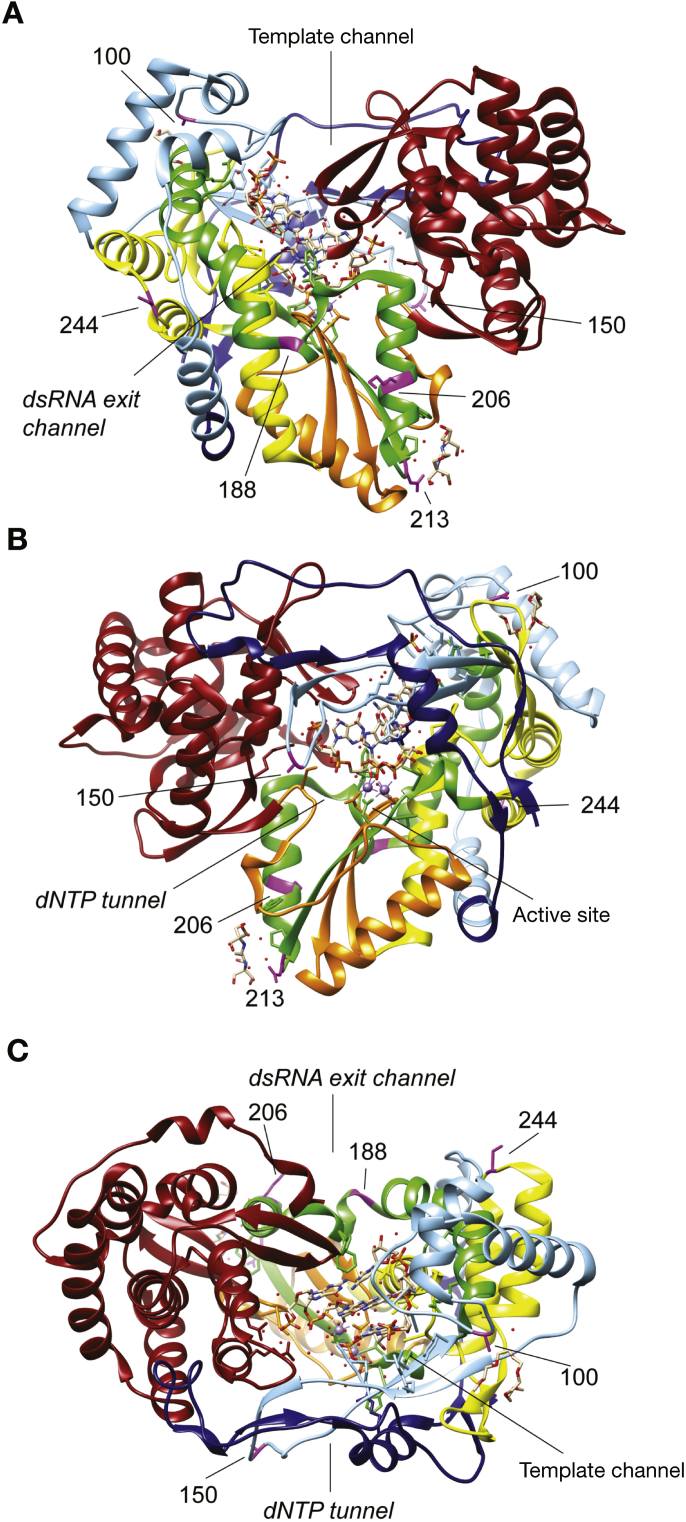
3-Dimensional model of the HCV polymerase NS5b (*A*, front; *B*, back; *C*, top) derived from the JFH-1 crystal structure with the location of the polymorphisms of interest shown in *purple*. The structure in the center represents a bound RNA strand. PDB crystal structure ID: 4WTG. Structure was annotated using the Chimera software. dNTP, deoxyribonucleotide triphosphate; dsRNA, double-stranded RNA.

To examine the structural significance of the observed polymorphisms, we plotted their location on a structural model of the HCV NS5b RNA polymerase. The crystal structure of the HCV genotype 3 NS5b has not been determined and we therefore used the well-established structure for strain JFH-1, a genotype 2a isolate.^23^ Most substitutions were located within domains I (“fingers”) and II (“palm”) of the protein (Figure 2). The palm domain contains the catalytic triad that forms the enzymatic active site. Highly conserved residues, crucial for the active site, are located at positions D220, D225, G317, D318, and D319.^24^, ^25^ While none of our identified positions are part of or adjacent to residues of the active site, 2 positions are in close proximity, K206E and T213N. The structural model shows that K206 (LYS-206) forms part of an α-helix close to the RNA binding cleft, but not directly within it. This residue is also close to a domain previously reported crucial for the RNA binding capacity of NS5b.^26^ Thr-213, however, is more distant from the catalytic site of the polymerase in the model.

The catalytic activity of NS5b is modulated by a direct interaction with NS5a.^27^, ^28^ This stimulates activity of the polymerase but can also inhibit activity in a dose-dependent manner.^27^ Specific domains on the NS5b are crucial for this interaction and include residues 143–145, 149–155, 365–371, and 382–388. A150V is located within one of these domains, which may affect NS5a binding and thereby potentially modulate the catalytic activity of NS5b. From a structural context, position 150 in NS5b is found within the finger domain—between the lysine-rich ends of the finger on a flexible loop that protrudes from the surface. This motif is highly conserved at its N- and C-termini, where a number of amino acids with positively charged side chains interact with incoming nucleotide triphosphates transiting to the active site and nucleotide binding pocket. This region is highly variable in sequence and likely structure. In the crystal structure of the closed conformation of the JFH-1 strain NS5b, residue 150 is on the end of a loop, protruding from the molecule over the nucleotide entry tunnel. Note that JFH-1 NS5b has a similar sequence in the finger-loop domain to genotype 3a. This loop is likely to be dynamic and not highly structured, as observed in flaviviruses and human immunodeficiency virus, where nucleotide interactions can trigger conformational changes in the finger domain, bringing it closer to the body of the polymerase toward the active site.^29^

### Effect of NS5b Polymorphisms on Drug Sensitivity

**Figure 3:**
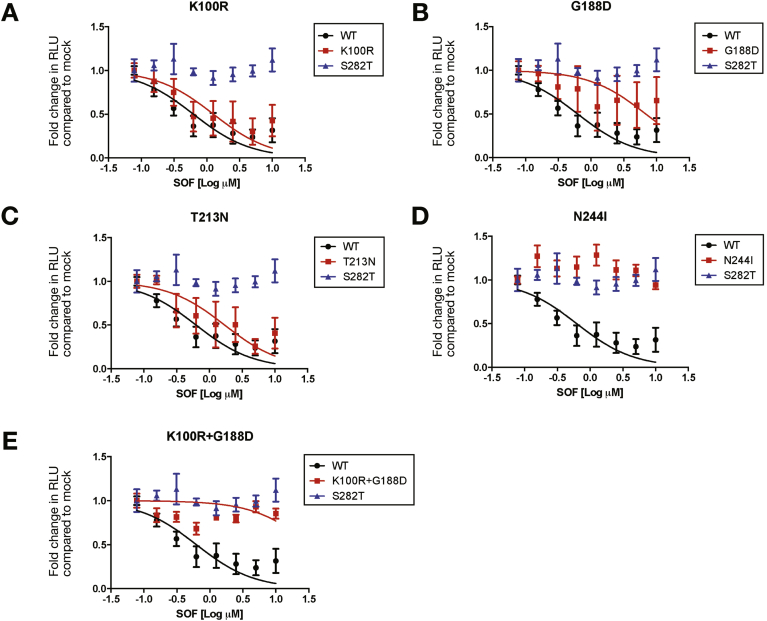
Huh7.5-SEC14L2 cells transiently transfected (by electroporation) with luciferase-containing HCV (S52-replicon) constructs with the NS5b polymorphisms from patient 9 (see Table 1). Cells were treated with SOF for 72 hours and replication was determined by luciferase assay, normalized to a sample 4 hours post electroporation for each construct. As a control for SOF non-response, a replicon containing the S282T NS5b mutation was included. *Panels A–E* indicate the effect of the polymorphisms from patient 9 to SOF sensitivity. IC_50_ values with 95% CIs were calculated by determining the drug concentration that affected a 50% reduction in HCV RNA and are summarized in Table 2. Mean values per drug concentration are plotted ± SEM. The above is representative of at least 3 independent experiments.

**Supplementary Figure 3:**
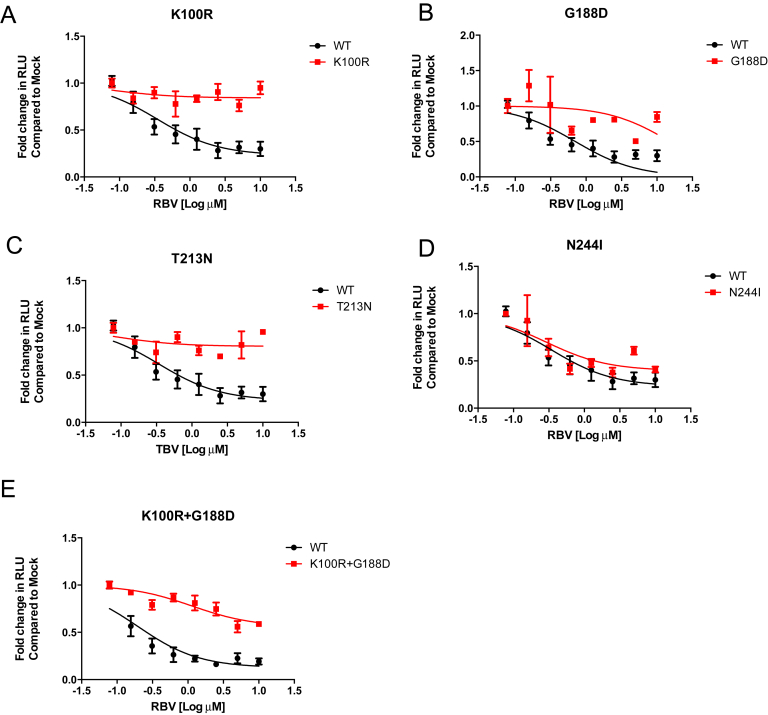
Huh7.5-SEC cells transiently transfected (by electroporation) with luciferase containing HCV (S52-replicon) constructs with the indicated NS5b polymorphisms were assessed. Cells were treated with RBV for 72 hours and replication was measured using a luciferase assay, normalized to a sample 4 hours post electroporation for each construct. *Panels A–E* indicate the effect of the polymorphisms from patient 9 to RBV sensitivity. The above is representative of at least 3 independent experiments. Relative luciferase units (RLU).

We examined the impact of the polymorphisms in modified HCV genotype 3 sub-genomic replicons. The identified polymorphisms were introduced into the S52 genotype 3 replicon^30^ and the nucleotide changes were confirmed by Sanger sequencing. The unique pattern of polymorphisms seen in patient 9 were associated with changes in sensitivity to SOF (Figure 3*A*–*E* and Table 2) and RBV (Supplementary Figure 3*A*–*E* and Supplementary Table 2). The largest impact on SOF sensitivity by an individual polymorphism was seen with N244I (a 16-fold increase in 50% inhibitory concentration [IC_50_] with non-overlapping CI). The K100R and T213N polymorphisms had a minimal impact on SOF sensitivity. The G188D polymorphism had a more marked effect (10-fold increase in IC_50_ with non-overlapping CI), but in combination with the K100R polymorphism, the effect on SOF sensitivity was increased and comparable to the level seen with the S282T variant. It should be noted that the combination of K100R and G188D and the N244I replicons had the greatest reduction in replication efficiency, as measured by relative luciferase (RLU) levels in comparison to the wild type (Wt). Attempts to generate viable replicons with further combinations of polymorphisms were not successful, precluding a full analysis of all of the variants and their interactions, but it is clear that combinations of mutations in HCV NS5b can modify the SOF response. Some of these polymorphisms (noticeably K100R) also reduced the effects of RBV. Given that patient 9 also had the Y93H NS5a polymorphism (Supplementary Table 1), known to modify the response to NS5a inhibitors, we speculate that multiple polymorphisms are required to influence the clinical response to short courses of SOF-based therapies. Intriguingly, patient 9 cleared the virus with a prolonged course of SOF/DAC and RBV therapy.

**Table 2:** 50% Inhibitory Concentration Values of Sofosbuvir Against Identified Hepatitis C Virus Genotype 3a Polymorphisms Compared With the Wild-Type S52 Replicon

S52 Replicon	SOF IC_50_ (95% CI), *μM*	Fold change in SOF IC_50_	Luciferase levels, *% of Wt*, mean ± SEM
Wt	0.63 (0.4–0.99)	1.00	100.00 ± 10.33
K100R	1.26 (0.64–2.5)	2.00	72.45 ± 13.18
A150V	4.88 (2.7–18.9)	7.75	98.17 ± 11.59
G188D	6.84 (2.47–18.9)	10.86	74.02 ± 17.11
K206E	5.25 (2.96–9.33)	8.34	59.35 ± 21.05
T213N	0.8 (0.85–3.43)	1.27	108.20 ± 18.19
N244I	10.32 (5.28–20.16)	16.38	55.95 ± 13.01
K100R+ G188D	19.91 (8.45–46.89)	31.80	40.96 ± 14.51
A150V + K206E	22.54 (12.47–40.74)	35.77	120.4 ± 17.75
S282T	45.15 (6.04–161.6)	71.67	67.85 ± 18.88

NOTE. IC_50_ values for SOF for each replicon construct are shown with 95% CIs. Fold-change in IC_50_ for SOF was calculated for each mutated construct using the unmodified Wt S52-replicon as a baseline. Relative luciferase levels compared to the Wt replicon construct were also included to measure the replication capacity of each replicon.

### Analysis of the A150V and K206E Polymorphisms

**Figure 4:**
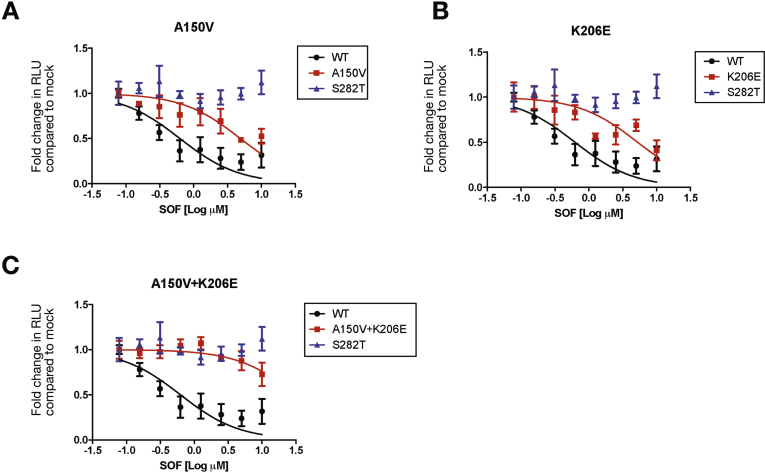
Huh7.5-SEC14L2 cells were transiently transfected (by electroporation) with luciferase containing HCV (S52-replicon) with Wt, 150V, and 206E, individually and in combination. Cells were treated with SOF for 72 hours and HCV replication was measured using a luciferase assay and normalized to a sample 4 hours post electroporation for each construct. *Panels A–C* indicate the effect of the polymorphisms (A150V and K206E) from patients 10*–*14 on SOF sensitivity. IC_50_ values with 95% CIs were calculated by determining the drug concentration that affected a 50% reduction in replication. Mean values per drug concentration are plotted ± SEM. The above is representative of at least 3 independent experiments.

**Supplementary Figure 4:**
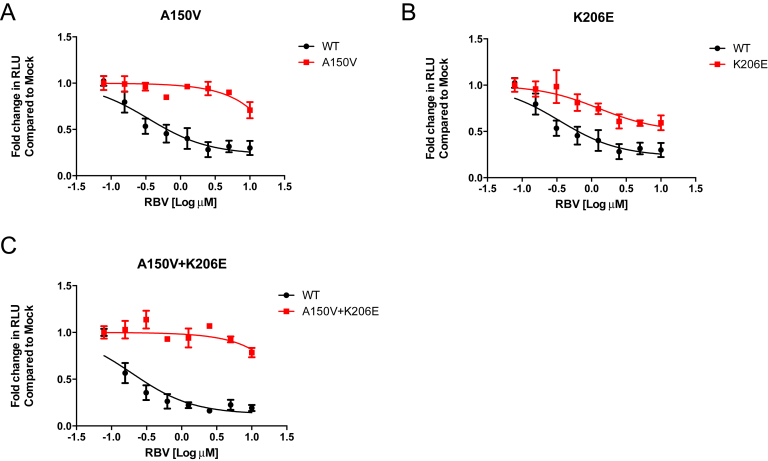
Huh7.5-SEC cells transiently transfected (by electroporation) with luciferase containing HCV (S52-replicon) constructs with the indicated NS5b polymorphisms were assessed. Cells were treated with RBV for 72 hours and replication was measured in a luciferase assay, normalized to a sample 4 hours post electroporation for each construct. *Panels A–C* indicate the effect of the polymorphisms from patients 10*–*14 on RBV sensitivity. The above is representative of at least 3 independent experiments. Relative luciferase units (RLU).

Examination of the common A150V and K206E polymorphisms in a transient replicon assay (Figure 4 and Table 2) individually showed a reduced sensitivity to SOF (8-fold) with non-overlapping CI. The combined polymorphisms (A150V and K206E) further reduced sensitivity to SOF (>35-fold) with non-overlapping CI. The effect of these polymorphisms on RBV sensitivity ranged from 10-fold for K206E to >40-fold change in IC_50_ for A150V, but in combination, the effect was enhanced (approximately 70-fold change in IC_50_ with non-overlapping CI) (Supplementary Figure 4 and Supplementary Table 2).

**Supplementary Figure 5:**
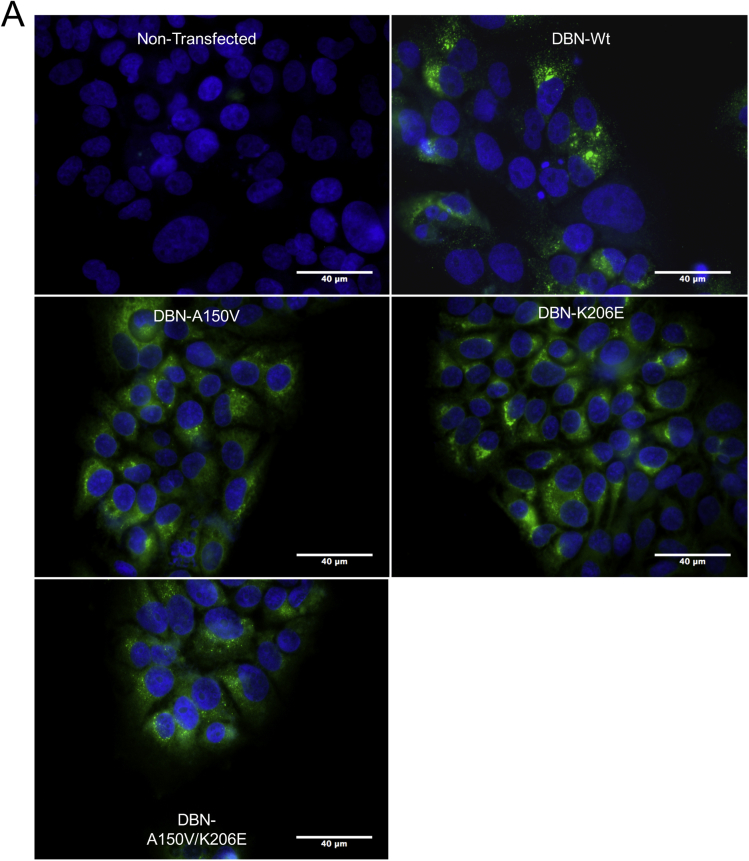
Huh7.5-SEC14L2 cells transfected with the viral constructs were stained for the HCV NS5a protein (*green*) by immunofluorescence 7 days post transfection to confirm establishment of replication complexes. Mock transfected cells were included as a negative control.

**Supplementary Figure 6:**
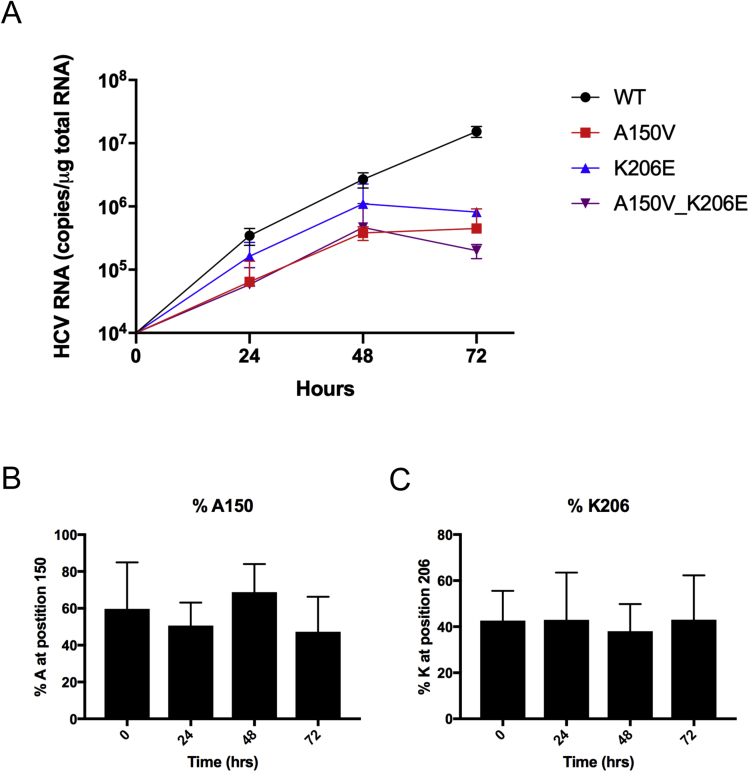
(*A*) Naïve Huh7.5-SEC14L2 cells were infected with Wt and DBN virus with the polymorphisms of interest at a multiplicity of infection of 0.05. Samples were taken at the indicated time points for quantification of HCV RNA by reverse-transcription quantitative polymerase chain reaction. Data presented are representative of 2 independent experiments and analyzed using a Mann-Whitney U test. Graph depicts mean ± SEM. (*B, C*) An equal inoculum of the Wt and mutant virus was used to infect the cells (multiplicity of infection = 0.03 median tissue culture infectious dose), the relative amounts of the competing viruses were determined by next-generation sequencing at 0, 24, 48, and 72 hours post infection and the percent of Wt (alanine at 150 or lysine at 206) is shown.

The transient subgenomic replicon assays demonstrated that NS5b polymorphisms A150V and K206E impact the sensitivity of the polymerase to both SOF and RBV. We sought to confirm our findings in an infectious HCV model, which covers all stages of the viral lifecycle and may be more representative of the situation in vivo. The A150V and K206E polymorphisms were engineered individually and in combination into the DBN3acc virus,^31^ and the presence of the substitution was confirmed by Sanger sequencing. Cells were transfected to generate virus stocks, and the presence of the DBN3acc was confirmed by immunofluorescence for NS5a (Supplementary Figure 5). The viral stocks generated were analyzed by NGS up to 26 days in culture and no nucleotide changes or new cell culture adaptations were observed (data not shown). The viral kinetics of the Wt virus and the polymorphisms A150V, K206E, and their combination were assessed over a 72-hour period. Up to 48 hours, the presence of polymorphisms did not have a significant impact on the infectivity, however, by 72 hours, a reduction in HCV RNA was observed in all viruses with the polymorphisms compared to the Wt (Supplementary Figure 6*A*). To further assess whether either the A150V or K206E polymorphism altered the viral fitness, the individual viruses were mixed with Wt virus at an equal ratio based on infectivity titer and used to infect naïve cells.^32^ Next-generation sequencing analysis of the infected cells over 72 hours demonstrated that the introduced polymorphisms did not confer a fitness advantage to the viruses, no virus dominated at any time point (Supplementary Figure 6*B–C*), suggesting that the observed changes in drug sensitivity were not due to enhanced viral replication.

**Figure 5:**
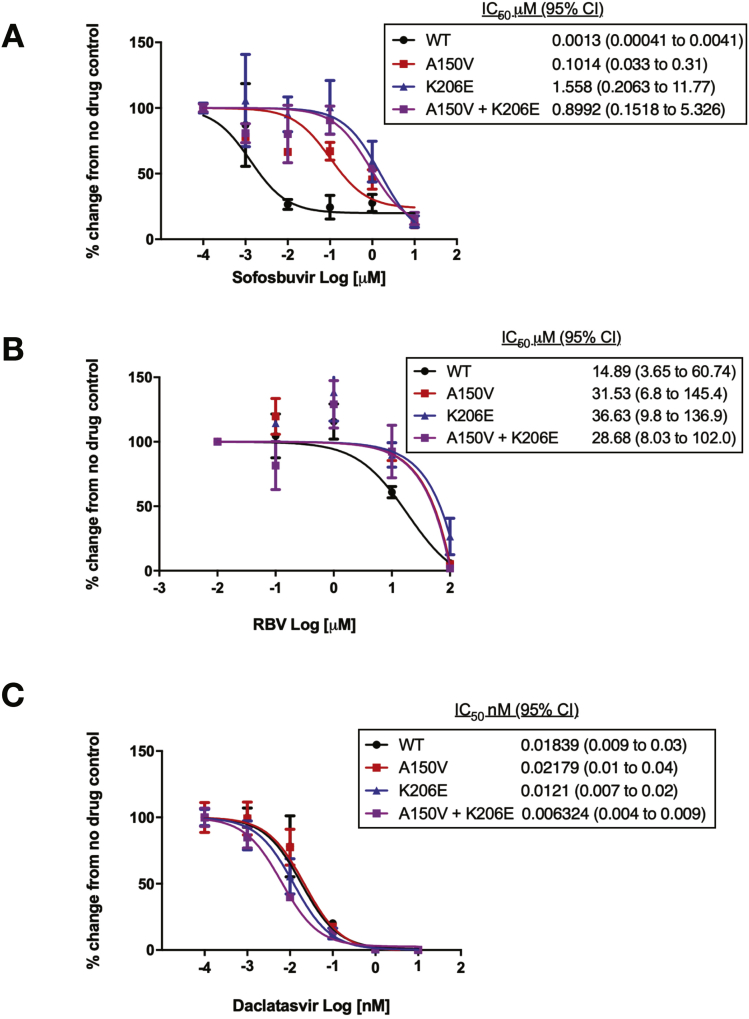
Sensitivity of HCV genotype 3 viruses to SOF, RBV, and DAC, comparison between the Wt and polymorphisms of interest. Huh7.5 SEC14L2 cells were infected with the indicated HCV genotype 3 viruses at an multiplicity of infection of 0.2 for 72 hours before treatment with serial dilutions of SOF (*A*), RBV (*B*), or DAC (*C*). After 24 hours of drug treatment, the cells were harvested (72 hours for RBV) and HCV quantified by reverse-transcription quantitative polymerase chain reaction. Concentration–response curves were calculated. IC_50_ values with 95% CIs were calculated by determining the drug concentration that affected a 50% reduction in HCV replication. Mean values per drug concentration are plotted ± SEM. Results show a mean of 2 independent experiments done in duplicate.

Next, we tested the sensitivity of our modified viruses to SOF, RBV, and DAC. We found that these viruses demonstrated a higher sensitivity to SOF than sub-genomic replicons yet the A150V and K206E mutations independently and in combination reduced the sensitivity to SOF (Figure 5*A*), confirming our previous findings. Additional reductions in sensitivity to RBV were also observed (Figure 5*B*), though these changes where not as pronounced as in previous replicon assays. Viruses were also assessed for their response to the NS5a inhibitor DAC (Figure 5*C*), no difference in sensitivity to either polymorphism, expressed individually or in combination in comparison to the Wt was observed. Together these data indicate that the polymorphisms A150V and K206E in the HCV NS5b polymerase individually and in combination cause a significant alteration in sensitivity to SOF in both replicon and infectious models.

## Discussion

SOF is a central component of some of the most effective drug regimens for the treatment of hepatitis C and a full understanding of its strengths and weaknesses will be important to ensuring it continues to be deployed appropriately. This is of particular importance, given the availability of alternative regimens for HCV genotype 3 that are based on the protease inhibitor glecaprevir.^33^ Studies on SOF “resistance” have been hampered by the extraordinary efficacy of the drug, which has led to very few treatment failures, although some very rare polymorphisms that can modify response, particularly in genotype 1 infection, have been identified.^34^ Viral resistance is normally identified by post-treatment emergence of variants that can be shown to reduce drug efficacy in laboratory studies. However, the marked variability of hepatitis C with multiple, rapidly evolving polymorphisms makes identification of “resistance-associated substitutions” difficult and very large cohorts are required. This is illustrated by the initial trial examining 8 weeks of SOF plus ledipasvir for 8 weeks,^35^ which did not suggest any impact of resistance-associated variants in the NS5a protein, but subsequent analysis of more than 2000 patients from multiple studies showed that variants in the NS5a protein modify response.^36^ It is thus very difficult to evaluate the mechanisms underlying a failure to respond to SOF-based regimens with classical approaches and with the increasing efficacy of combining SOF with a protease inhibitor and a NS5a inhibitor it is likely that identifying motifs associated with failure will require massive patient cohorts. It is believed that SOF does not generate significant viral resistance and can therefore be used successfully for retreatment regimens. This hypothesis has been confirmed by clinical studies in which patients who failed to respond to SOF were successfully retreated with a combination of SOF, velpatasvir, and voxilaprevir, although a tiny number of patients did not achieve a virologic response,^8^ and this was most marked in patients with genotype 3 HCV and cirrhosis who had failed to respond to an NS5a-containing regimen, where response rates were 93%. Given the difficulties of identifying SOF “resistance” by conventional sequence-based assays, we have adopted a phenotyping approach that has allowed us to examine previously unexplored viral variants that impact on the response to SOF.

SOF is a potent pan-genotypic antiviral drug, but patients infected with genotype 3 HCV respond less well than other genotypes, and response to therapy is further reduced by the presence of cirrhosis or previous failure to respond to IFN. We studied patients who failed to respond to SOF-containing regimens that are now known to be inadequate. Our analysis shows that reduced response to SOF may occur if multiple viral polymorphisms are combined.

The strength of this study is the use of multiple different phenotypic analyses on viral variants of interest coupled with a genetic analysis in a cohort of patients who received SOF-only therapies without potent NS5a inhibitors. The use of different assay systems (capture-fusion replication, viral replicons, and modified viruses) showed that different assays have different characteristics and the efficacy of SOF in vitro is heavily dependent on the assay deployed.

We do note that specific IC_50_ values for antiviral drugs tested in our assay against the recently characterized DBN3acc virus differ from those published previously,^31^ we attribute this difference to our use of RNA copies to measure viral response as opposed to quantifying cells positive for viral proteins. We found some reduction in replication efficiency in the modified replicons, however, the maximum changes were of a similar magnitude to those seen with the S282T variant,^13^ and we therefore do not believe that they significantly alter our conclusions that these mutations impair the efficacy of SOF. Previous studies suggest that an increase in replicative fitness can modify antiviral sensitivity,^32^ but as the modified viruses we generated in this study demonstrated no significant increase in replicative fitness, we can exclude this as an explanation for the observed changes in drug sensitivity.

Collectively, our observations may help to explain why SOF polymorphisms that emerge during therapy do not appear to affect the sensitivity to SOF in vitro.^13^, ^15^ Indeed, recent studies that modeled SOF resistance in vitro identified variants that had a significant impact on viral sensitivity to SOF, but were rarely found in clinical isolates.^31^ It is unclear which of the different assays most accurately reflects the conditions in patients, but in this study we found a very consistent pattern of response—in all of the assays, the same polymorphisms had similar effects, although the magnitude differed quite markedly in the different assays. The main weakness of this study is the use of samples from treatment regimens that have been superseded by more-potent drug combinations. However, treatment failure with the new, triple-therapy regimens is rare, and an analysis of the outcomes of such treatments will require very large, real-world cohorts that are only now beginning to emerge.

Clinically, our data show that unusual viral polymorphisms can impair the response to SOF. For treatment-naïve patients, responses to SOF-based treatments are so effective that any impact of these viral variants is likely to be minimal, and we would not recommend that such patients undergo pretreatment “resistance” testing. For patients who have been exposed to multiple drug regimens (including IFN and NS5a inhibitors, which can lead to resistance-associated polymorphisms), we speculate that the polymorphisms identified here might be of significance, and we suggest that pretreatment viral sequencing may be useful in selecting the optimal regimen for such patients. Further studies in large cohorts of patients exposed to diverse treatment regimens will no doubt identify the small cohorts of patients who require sophisticated virologic analysis before therapy.

## Figures and Tables

**Supplementary Table 1:** Details of clinical samples used in the capture-fusion phenotyping assay

Patient no.	Therapy	Outcome	SOF/RBV sensitivity	Age, *y*	Sex	Cirrhosis	Previous IFN treatment	Baseline viral load, *IU/mL*	NS5A RAS, baseline
1	SOF/DAC/RBV	SVR	Sensitive	59	M	Decompensated	Yes	2,178,912	—
2	SOF/VEL	SVR	Sensitive	32	F	Compensated	No	5,992,729	—
3	Ombitasvir/paritaprevir/ritonavir+SOF	SVR	Sensitive	61	F	Non-cirrhotic	Yes	2,301,777	—
4	SOF/DAC	SVR	Sensitive	59	F	Non-cirrhotic	No	2,663,854	—
5	SOF/LDV/RBV	Relapse	Sensitive	47	M	Decompensated	No	2,321,679	—
6	SOF/DAC/RBV	Relapse	Sensitive	49	M	Decompensated	Yes	1,913,455	—
7	SOF/LDV/RBV	Relapse	Sensitive	52	M	Decompensated	Yes	808,776	—
8	SOF/LDV	Relapse	Sensitive	49	M	Decompensated	No	3,100,000	—
9	SOF/DAC/RBV	Relapse	Insensitive	48	M	Decompensated	No	1,585,601	Y93H
10	SOF/LDV/RBV	Relapse	Insensitive	54	M	Decompensated	No	255,934	—
11	SOF/LDV/RBV	Relapse	Insensitive	56	M	Non-cirrhotic (transplant graft)	Yes	2,362,045	—
12	SOF/DAC/RBV	Relapse	Insensitive	58	M	Decompensated	Yes	902,000	—
13	SOF/LDV/RBV	Relapse	Insensitive	43	M	Decompensated	No	5,670,819	NA
14	SOF/LDV/RBV	Relapse	Insensitive	50	M	Decompensated	Yes	570,000	Y93H

NOTE. Details of the samples used in the capture fusion assay (Figure 1 and Table 1). Data include treatment, clinical outcome, age, sex, disease status, and baseline viral load.

F, female; LDV, ledipasvir; M, male.

**Supplementary Table 2:** 50% Inhibitory Concentration Values of Ribavirin Against Identified Hepatitis C Virus Genotype 3a Polymorphisms Compared With the Wild-Type S52 Replicon

S52 replicon	RBV IC_50_ (85% CI), μM	Fold-change in RBV IC_50_	Luciferase levels, *% of Wt*, mean ± SEM
Wt	0.70 (0.47–1.05)	1.00	100.00 ± 10.33
K100R	23.61 (9.96–55.95)	32.85	72.45 ± 13.18
A150V	28.03 (17.14–45.83)	40.04	98.17 ± 11.59
G188D	15.19 (4.94–46.67)	21.70	74.02 ± 17.11
K206E	7.40 (4.66–11.75)	10.57	59.35 ± 21.05
T213N	6.12 (3.58–10.54)	8.74	108.20 ± 18.19
N244I	1.49 (0.78–2.86)	2.13	55.95 ± 13.01
K100R+G188D	7.27 (5.15–10.25)	10.35	40.96 ± 14.51
A150V+K206E	48.69 (20.81–113.9)	69.55	120.4 ± 17.75

NOTE. The calculated IC_50_ values with 95% CIs and the fold-change compared to the Wt replicon from Supplementary Figures 3 and 4 were replicons were treated with RBV, are summarized. Relative luciferase levels compared to the Wt replicon construct for each polymorphism were also included.

## Supplementary Methods

### Cells and Viral Phenotyping

Huh7.5-SEC14L2 cells (kind gift from P. Simmonds, Oxford, UK) were propagated in Dulbecco's modified Eagle's medium supplemented with 10% fetal calf serum. ThP1 cells were propagated in RPMI supplemented with 10% fetal calf serum. Cytokines used for ThP-1 stimulation were phorbol 12-myristate 13-acetate (Sigma-Aldrich, Dorset, UK) and interferon gamma (Invitrogen, Paisley, UK). SOF was kindly provided by Gilead Sciences (Foster City CA), DAC by Bristol-Myers Squibb (New York, NY), and RBV by Sigma Aldrich (St Louis, MO). All antiviral drugs were reconstituted in dimethyl sulfoxide.

Viral phenotyping was performed using the capture-fusion viral replication assay, as described previously.^1^ Briefly, THP-1 cells were seeded into 6-well plates (10^6^ cells/mL) and maintained for 18 hours, with or without the addition of IFN gamma (10 ng/mL) and phorbol 12-myristate 13-acetate (200 ng/mL). Cells were washed 3 times and the medium replaced with RPMI/2% fetal calf serum and patient serum (1 HCV IU/cell). After incubation at 37°C for 18–24 hours, the supernatant was removed and cells were washed. Adherent cells were removed using a cell scraper and Huh7.5 cells added at a 1:1 ratio. Cell fusion was performed as above. The fused cells were seeded into 6-well plates at a density of 10^5^ cells/mL and maintained at 37°C in the presence or absence of antiviral drugs for 3 days.

HCV RNA was quantified by reverse-transcription polymerase chain reaction (PCR). Total RNA was extracted with TRIzol (Invitrogen) and quantified using RiboGreen (Invitrogen), according to the manufacturer’s instructions. One-step reverse transcription PCR (QuantiTect Virus Kit Qiagen, Crawley, UK) and TaqMan Gene Expression Assay HCV primer and probe (Applied Biosystems, Paisley, UK) were used for quantification. PCRs were performed in triplicate. Absolute quantification of HCV RNA was performed by including serial dilutions of an RNA standard in each PCR run and expressed relative to total sample RNA.

To assess cytotoxicity of antiviral drugs, cells were seeded in a 96-well plate in triplicate and incubated in the presence of a serial dilution of drugs for 72 hours. A 10X AlamarBlue reagent was added directly to the cell media and incubated for 3 hours at 37°C. Fluorescence at excitation wavelength of 560 nm and emission wavelength of 590 nm was then quantified.

### Sequencing

Sequencing of patient samples by Metagenomic and Target Enrichment was performed after RNA extraction from 200 μL plasma (Agencourt RNAdvance Blood kit; Beckman Coulter, Brea, CA), eluted into 11 μL water, and reverse transcribed (SuperScript III; Invitrogen) with random hexamers and a Second Strand Synthesis kit (New England Biolabs, Ipswich, MA). Library preparation was performed with the KAPA Library Prep kit (KAPA Biosystems, Wilmington, MA) with index tagging by 16 cycles of PCR using KAPA HiFi HotStart (KAPA Biosystems) and custom-synthesized multiplex oligos. Libraries were quantified by Qubit (ThermoFisher, Waltham, MA) and TapeStation (Agilent), pooled at equimolar concentrations for sequencing on the Illumina MiSeq (v3 chemistry; Illumina, San Diego, CA). For target enrichment, pooled libraries were enriched by the NimbleGen SeqCap EZ System (Roche, Basel, Switzerland), followed by sequencing on the Illumina MiSeq platform using v3 chemistry. Variant analysis was performed after Fastq file quality assessment using FastQC and Sam files were created by mapping against whole-genome HCV reference sequences. Assemblies were viewed using UGene and positions of interest identified in the GenBank HCV strain H77 polyprotein gene (accession no. AF011751). Each reference was aligned against HCV strain H77 to standardize the positions of interest and total base counts were identified at the variant positions.

### Hepatitis C Virus Target Enrichment

RNA was isolated from 200 μL of cell culture supernatant using the RNAdvance Blood extraction kit (Beckman Coulter) and collected in 27 μL water. After conversion of RNA to double-stranded DNA, libraries were prepared for Illumina sequencing using the KAPA DNA LTP Library Preparation Kit (Roche), and NEBNext Multiplex Oligos for Illumina (New England Biolabs). Libraries were quantified using Qubit dsDNA HS Assay Kit (Invitrogen) and size distribution assessed using Agilent TapeStation with D1K High Sensitivity Kit (Agilent); libraries were normalized accordingly to viral load and mass. A 500-ng aliquot of the pooled library was enriched using SeqCap EZ Developer Probes (Roche) following manufacturer’s protocol. After a 14-cycle post-enrichment PCR, the cleaned pool was sequenced with 151 base-paired end reads on an Illumina NextSeq cartridge, with resulting reads characterized by Q30 >87%.

### Site-Directed Mutation Primers

Outer NS5b primers encoding XhoI and XbaI restriction sites at the 5′ and 3′ end of NS5b

S52-5b XhoI F TGCCTCCTCTCGAGGGAGAG

S52-5b XbaI R GGTCGACTCTAGACATGATCTGC

Overlapping primers designed to introduce the specified amino acid change in NS5b

S52-5b-K100R-F GTCCCTCCTCACTCTGCCCGGTCGAGGTTCGGGTATAGTGCGAAGG

S52-5b K100R-R CCTTCGCACTATACCCGAACCTCGACCGGGCAGAGTGAGGAGGGAC

S52-5b-G188D-F GTCAATTGAGACGATGGATTCCGCTTATGGATTCCAATACTC

S52-5b-G188D-R GAGTATTGGAATCCATAAGCGGAATCCATCGTCTCAATTGAC

S52-5b-A150V-F GTTTTGTGTGGACCCCGTTAAAGGGGGCCGC

S52-5b-A150V-R GCGGCCCCCTTTAACGGGGTCCACACAAAAC

S52-5b-K206E-F CGGGTCGAACGTCTACTGGAGATGTGGACCTCAAAG

S52-5b-K206E-R CTTTGAGGTCCACATCTCCAGTAGACGTTCGACCCG

S52-5b-T213N-F GAAGATGTGGACCTCAAAGAAAAACCCCTTGGGGTT

S52-5b-T213N-R AACCCCAAGGGGTTTTTCTTTGAGGTCCACATCTTC

S52-5b-N244I-F GGAGATATATCAATGCTGTATCCTTGAACCGGAGGC

S52-5Bb-N244I-R GCCTCCGGTTCAAGGATACAGCATTGATATATCTCC.

### Immunofluorescence

Cells were plated onto glass cover slips at 1 × 10^4^/cm^2^ 12–14 hours before experiment. Cells were then washed with phosphate buffered saline and fixed in ice-cold methanol at –20°C for 20 minutes and then washed in phosphate buffered saline and blocked with 3% fetal bovine serum for 1 hour at room temperature. The sheep polyclonal anti-NS5a (kind gift from Mark Harris, Leeds University) was diluted in blocking buffer at 1:1000 and cells were incubated for 1 hour at room temperature. Unbound primary antibody was removed by 3 washes with 1X phosphate buffered saline before incubation with Alexa Flour 488 donkey anti-sheep secondary antibody diluted to 1:1000 for 1 hour at room temperature. Cells were then incubated with 4′,6-diamidino-2-phenylindole (1:1000) for nucleus visualization.

### Relative Fitness Assay

Growth-competitions experiments were established between the DBN_WT and DBN_A150V or DBN_K206E in Huh7.5 cells. Huh7.5 cells (4 × 10^5^) were infected with a 1:1 mixture of the 2 competing viruses, based on their infectivity titers (total, 1.2 × 10^4^ median tissue culture infectious dose: multiplicity of infection of 0.03 median tissue culture infectious dose/cell), following Sheldon et al.^2^ RNA was extracted from the initial mixture (0 hours) and from cells at 24, 48, and 72 hours post infection. PCR products were generated for the regions of interest with the addition of common sequence tags CS1 and CS2 to allow for the addition of a sample index barcode.

A150V-F GACACCACAACCCCAATTCCAACTACCATAATGGCG,

A150V-R CACGTCATATAGGGCACGTTTCTCACAGACACGCACCCCC,

K206E-F GTCTGCCTACGGATTCCAATACTCGCCTCAACAGCG,

K206E-R CTGTTCAGTGACAGTCGAGTCAAAGCAGCGGGTGTC

Fastq files were decompressed and the grep command line tool used to perform a count analysis on each sample to determine the number of counts for each variant. A flanking region was used upstream and downstream of the variant site to ensure high sequence similarity. The following sequences were used:

A150V-alanine: GACCCCGCTAAGGGG

A150V-valine: GACCCCGTTAAGGGG

K206E-lysine: CTGCTGAAGATGTGG

K206E-glutamic-acid: CTGCTGGAGATGTGG

Counts for each sample were tabulated and a percentage of the total number of reads calculated. Any sample with <15 reads in total was considered a fail and these data were not reported.
